# Multifunctional Iron Bound Lactoferrin and Nanomedicinal Approaches to Enhance Its Bioactive Functions

**DOI:** 10.3390/molecules20069703

**Published:** 2015-05-26

**Authors:** Jagat R. Kanwar, Kislay Roy, Yogesh Patel, Shu-Feng Zhou, Manju Rawat Singh, Deependra Singh, Muhammad Nasir, Rakesh Sehgal, Alka Sehgal, Ram Sarup Singh, Sanjay Garg, Rupinder K. Kanwar

**Affiliations:** 1 Nanomedicine-Laboratory of Immunology and Molecular Biomedical Research (NLIMBR), School of Medicine (SoM), Molecular and Medical Research (MMR) Strategic Research Centre, Faculty of Health, Deakin University, Waurn Ponds, Victoria 3217, Australia; E-Mails: k.roy@deakin.edu.au (K.R.); yogeshpatel3545@gmail.com (Y.P.); rupinder.kanwar@deakin.edu.au (R.K.K.); 2 Department of Pharmaceutical Sciences, College of Pharmacy, University of South Florida, Tampa, FL 33612, USA; E-Mail: szhou@health.usf.edu; 3 University Institute of Pharmacy, Pt. Ravishankar Shukla University, Raipur 492 010, India; E-Mails: manjursu@gmail.com (M.R.S.); deependraiop@gmail.com (D.S.); 4 Department of Food Science & Human Nutrition, Faculty of Bio-Sciences, University of Veterinary & Animal Sciences, Lahore, Punjab 54000, Pakistan; E-Mail: nasir@uvas.edu.pk; 5 Department of Medical Parasitology, Postgraduate Institute of Medical Education & Research, Chandigarh 160012, India; E-Mail: sehgalpgi@gmail.com; 6 Department of Obstetrics & Gynecology, Government Medical College & Hospital, Sector 32, Chandigarh 160031, India; E-Mail: alkasehgal@rediffmail.com; 7 Carbohydrate and Protein Biotechnology Laboratory, Department of Biotechnology, Punjabi University, Patiala 147002, India; E-Mail: rssingh11@lycos.com; 8 Centre for Pharmaceutical Innovation and Development (CPID), School of Pharmacy and Medical Sciences, University of South Australia, Adelaide SA 5000, Australia; E-Mail: sanjay.garg@unisa.edu.au

**Keywords:** lactoferrin, camel, bovine, immunity, cancer, nanoparticles

## Abstract

Lactoferrin (Lf), an iron-binding protein from the transferrin family has been reported to have numerous functions. Even though Lf was first isolated from milk, it is also found in most exocrine secretions and in the secondary granules of neutrophils. Antimicrobial and anti-inflammatory activity reports on lactoferrin identified its significance in host defense against infection and extreme inflammation. Anticarcinogenic reports on lactoferrin make this protein even more valuable. This review is focused on the structural configuration of iron-containing and iron-free forms of lactoferrin obtained from different sources such as goat, camel and bovine. Apart for emphasizing on the specific beneficial properties of lactoferrin from each of these sources, the general antimicrobial, immunomodulatory and anticancer activities of lactoferrin are discussed here. Implementation of nanomedicinial strategies that enhance the bioactive function of lactoferrin are also discussed, along with information on lactoferrin in clinical trials.

## 1. Introduction

Milk proteins are considered to be the most important source of bioactive peptides, as an increasing number of bioactive peptides have been identified in milk protein hydrolysates. The potential health benefits of milk protein-derived peptides have been a subject of growing commercial interest in the context of health-promoting functional foods [1]. Lactoferrin (Lf) belongs to the transferrin (Tf) family and is a non-heme iron binding glycoprotein with molecular weight of 78 kDa that contains around 690 amino acid residues. It is found in bovine milk as well as in humans [2]. In humans, it is one of the major proteins of all exocrine secretions including saliva, tears, semen, vaginal fluids, gastrointestinal fluids, nasal mucosa and bronchial mucosa [3,4]. Breast milk represents the main source of Lf found in the gut of infants and high levels of fecal Lf in in the first days of life represents the initiation, development and/or composition of the neonatal gut microbiota [5]. Lf is also known for its anti-bacterial, antifungal, antiviral, antimicrobial, anti-oxidant, anti-inflammatory, antiparasitic, anti-allergic and most importantly anticancerous properties [3,6]. The highest concentration of lactoferrin is found in human colostrum and then human milk followed by cow milk, and it is the second most abundant milk protein after Caseins [7]. This natural protein is proving to be a highly promising biodrug in anticancer research due to its potential use as a natural agent for combating cancer. The use of chemotherapeutic agents has posed a major risk of failure due to the development of drug-resistant cancers. This limitation demands the need of a natural molecule that has patient compliance and can possibly completely eradicate the primary tumor, thus eliminating the risk of recurrence.

Lactoferrin: Structure and Functions

**Figure 1:**
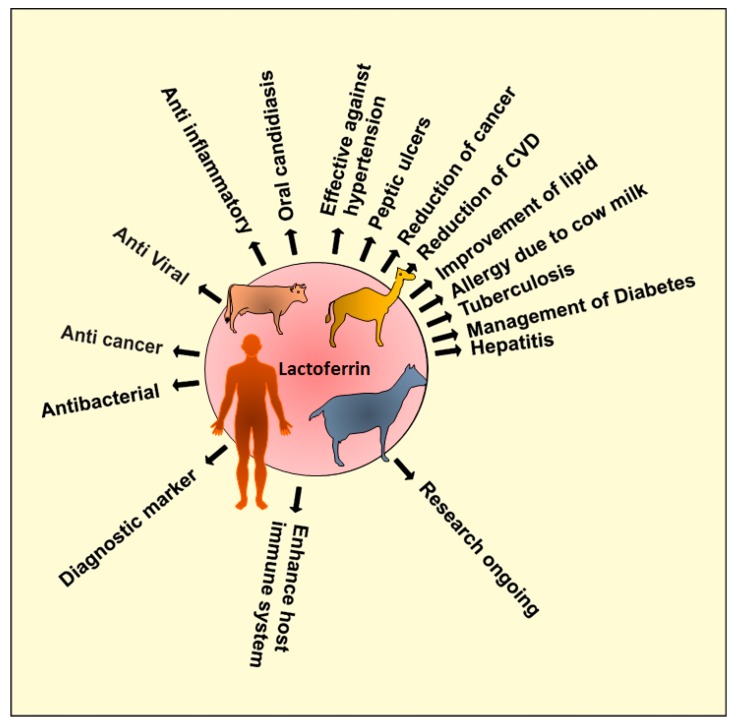
Lactoferrin and its functions. Lf from bovine source is a promising candidate as an anticancer agent [13,14]. Lf from goat source is still unexplored and not many studies have been conducted to determine its unknown therapeutic efficiency [15]. Lactoferrin is the focus of the recent interest. Lf from camel was found to be more effective against hepatitis [16] and diabetes [17], thus proving to be a broad spectrum therapeutic, antibacterial [18] protein. A very striking study on human lactoferrin has shown that it can be used as a diagnostic marker for various cancers. Moreover, Lf also boosts the host immune response [19,20] and is also known to have an anti-inflammatory activity [21].

The structure of Lf consists of a single polypeptide chain which is folded into two lobes (N and C lobes) with 33%–41% homology [8]. Both lobes are linked by an α-helical residue, making Lf a flexible molecule. The two lobes of Lf are made of α-helix and β-sheet, and each lobe can bind either Fe^+2^ or Fe^+3^ ions in synergy with the carbonate ion (CO_3_^2−^) [9]. The iron binding affinity of Lf is known to be the maximum amongst transferrin family. Lf can remain bound to iron in varying pH range [10]. Distribution of positive charges at the *N* terminus (1–7), the first helix (13–30), and in the inter lobe region is one of the specific features of Lf [2]. There are two forms of Lf, namely the iron-free form (Apo-Lf) and the iron containing (holo-Lf) [2]. Lactoferrin (Figure 1) is considered to be an important host defense molecule and has a diverse range of physiological functions such as antimicrobial/antiviral activities, immunomodulatory activity, and antioxidant activity [11,12].

The major role of Lf in humans is the transportation of iron in blood plasma [9]. Lactoferrin, in its natural form, is partially saturated with iron and hence can be fully saturated with iron from the external environment [13,22]. It has been reported that lactoferrin regulates multiple signaling pathways to impart cytotoxic effects on cancer cells. Bovine lactoferrin (bLf) and human lactoferrin (hLf) can induce cytotoxic effects in cancer cells by inducing a cell cycle arrest leading to apoptosis, while bovine lactoferricin B inhibits cell growth by triggering mitochondrial-related apoptosis (intrinsic apoptotic pathway) and disrupting the cell membrane. All the three forms are known to inhibit the activity of Akt (protein kinase B), survivin and to activate p21, p27, p38, and JNK (c-Jun *N*-terminal kinase) and to induce the release of caspase-8, caspase-3, and cytochrome c to induce apoptosis [14,23].

## 2. Sources of Lactoferrin

Lf is an important part of the innate immune system [24]. Lf is continuously synthesized in body and is released into the exocrine fluids like saliva [25], tears [26] and vaginal fluids [27], or only at well-defined stages of cell differentiation such as, the granules of neutrophils [28]. Glandular epithelial cells secrete Lf in milk source. Various concentrations of Lf is found in the milk obtained from different sources [29]. During an infection or an inflammatory condition, the levels of Lf are raised in the body [30] making Lf a biomarker for inflammatory conditions. Lf has been found to have a therapeutic potential and for this reason there have been several attempts to isolate Lf from various sources (Table 1).

**Table 1:** Lactoferrin: various sources, functions and roles.

Lactoferrin Source	Action	Functional Role	Reference
Human Lactoferrin	Anti-microbial	Effective against *Streptococcus*, *Salmonella*, *Shigella*, *Staphylococcus* and *Enterobacter*.	[31–34]
		Enhances the host immune system.	
	Anti-cancer	Diagnostic marker.	
Goat Lactoferrin	Ongoing research	Still novel and further studies need to be conducted	[15]
Camel Lactoferrin	Anti-viral	Inhibits infection by Hepatitis C and B virus. It has hepatoprotective effect.	[16,17,35,36]
	Anti-diabetic	Potential therapeutic molecule in targeting both type 1 and type 2 diabetes. More work needs to be done.	
Bovine Lactoferrin	Anti-cancer	Anticancer activity against colorectal cancer and lung cancer.	[13,14,18,21,37,38]
	Anti-microbial	Effective against oral candidiasis, influenza virus pneumonia and skin infections due to herpes virus.	
		Enhances host immune response	
		Anti-inflammatory.	

Lf regulates inflammatory cytokines production in a mode resembling to other anti-inflammatory cytokines by suppressing inflammation interacting with macrophages and restraining the production of inflammatory cytokines by cells [39,40]. Lf is known to suppress the production of TNF-α, IL-1β, IL-6 and IL-8 in human mononuclear cells (*in vitro)* [41] and improve production of IL-10 and IL-4 (*in vivo*) [42] which explain its ability to reduce β-cell destruction.

### 2.1. Human Lactoferrin

Human Lf (hLf) is isolated from the colostrum by various methods including chromatographic techniques like ion exchange chromatography (ref). Saturated forms of Lf were prepared by dialysis [43]. hLf was tested against a number of bacteria to observe its bactericidal activity and it was reported that Lf exhibited a very effective response against a various range of bacteria including species of *Streptococcus*, *Salmonella*, *Shigella*, *Staphylococcus* and *Enterobacter* [31]. An important aspect of hLf is its role in inhibiting the growth of solid tumours in mice. The anti-cancer potential of hLf was investigated on the B16-F10 melanoma cells, and it was observed that treatment with Lf inhibited colonization of the tumour in the lungs. Lf also activated the natural killer (NK) cells, enhanced antibody dependent cell cytotoxicity and increased the production of macrophages [32]. hLf has also been found to play a role in the nutritional activity by increasing the thymidine content in damaged crypt cells helping in their recovery and development [33]. hLf is also used as a diagnostic marker as its immunochemical detection in the feces indicates the presence of gastrointestinal disorders and risk of colon cancer [34].

### 2.2. Goat Lactoferrin

A very novel source of Lf, goat Lf has been studied after the beneficial effects of bLf. The concentrations of lactoferrin in goat milk during various stages of lactation have been researched by a group at the University of Bonn in Germany. They proved that the concentration of Lf was directly proportional to the number of somatic cells in the samples, both of which are influenced by a number of physiological parameters in the body [15]. In another study, the glycosylation of goat milk Lf was compared with that of human and bovine milk glycoproteins. The data obtained using Nano-LC-Chip-Q-TOF MS identified 65 structures, including high mannose, hybrid, and complex N-glycans. These results not only demonstrated the presence of analogous glycans in human and goat milk but also identified novel glycans in goat milk that were not present in human milk [44]. Therefore, goat milk is also a possible candidate for infant formula supplementation as it shares high homology with human lactoferrin.

### 2.3. Camel Lactoferrin

Camel milk is very popular amongst several traditional medicines. For example, it is used for hepatitis in Egypt [45]. Studies have indicated that the camel milk stimulates the immune response in hepatitis B patients. It helps to strengthen the cellular immune response by inhibiting the replication of viral DNA and thus promoting the recovery of the chronic hepatitis B patients. Camel Lf plays a key role as an antiviral agent against HBV through interaction with heparin sulfate on the cell surface, which appears to obstruct the virus attachment [16]. Camel Lf also exhibits its activity against HCV inhibiting entry into human leukocytes and HepG2 cells [35,45].The potential activity of camel Lf against HCV in HepG2 and lymphocytes is also reported by El-Fakharany *et al.* [45]. Camel Lf inhibits HCV entry and replication inside the human peripheral blood and HepG2 through direct interaction with virus *in vitro*. Bovine Lf can be used as therapeutic for chronic hepatitis C along with interferon [36], however camel Lf seems to be effective without an adjunct therapy. The carboxyl region of Lf that has 33 amino acid sequences similar to human CD81 is supposedly responsible for binding HCV envelop protein. Moreover, hepatic iron overload due to HCV infection increases reactive oxygen species (ROS) production that might initiate lipid peroxidation, steatosis and depletion of glutathione stores, acceleration in hepatic injury resulting in hepatocellular necrosis/carcinoma [46,47]. Camel Lf exhibits dual function; inhibits lipid peroxidation and regulates the hepatic iron content by the aptitude to behave, unlike other Lf and Tf, as half Lf “iron binding protein” and half Tf “iron transporter protein” [48,49].

The bioactive components present in camel milk include lactoferrin, lactoperoxidase, lysozyme and insulin like protein [50]. Camel Lf, also isolated from the colostrum of camel milk, is the first protein of the superfamily Tf that displays characteristics of iron binding. Iron release capacity of camel Lf is a pH dependent process [48]. Camel Lf also improves the imbalance of Th1/Th2 cytokine that occurs during any hepatocyte damage exhibiting hepatoprotective effect [16,51].

In a study conducted by National Research Center at Cairo, it was found that like human and bovine Lf, camel Lf does not prevent the entry of HCV into the host rather, on its interaction with HCV, it leads to complete inhibition of the virus within 7 days of incubation [35].

The advantages of camel Lf are not limited to hepatic disorders. Lf acts as anti-oxidants by minimizing the damage induced by aluminium chloride and cadmium chloride by improving the production of antioxidant enzymes [52]. The role of camel Lf is still novel and needs to be taken forward to look at its activity in order to target microbes, inflammation, and cancer.

Milk from camel also improves the β-cell functions and has a hypoglycaemic effect due to reduced β-cell work overload, increase in tolerance to insulin levels and presence of insulin like factor [17]. The reduction in β-cell destruction is due to a reduction in the levels of inflammatory cytokines along with regulation of the immune system and regulatory cells.

### 2.4. Bovine Lactoferrin

The concept of oral administration of bovine lactoferrin (bLf) was first introduced in 1978 when a bLf containing dry milk was marketed by the Morinaga Milk industry in Japan [53]. Later on, the research and evidence have indicated the role of orally administered bLf in the improvement in intestinal microbial flora, increased serum ferritin and hematocrit levels [54], reduction in lower respiratory track diseases and anti-infective activities [55]. Orally administered bLf has also shown beneficial effects in other animal infection models including oral candidiasis [56], influenza virus pneumonia [57] and skin infections due to herpes virus [58]. Enhanced production of interleukin-18 (IL-18) in intestinal epithelial cells, IL-10 and interferon-γ (IFN-γ) in intestinal intraepithelial lymphocytes and mesenteric lymph node cells, CD4^+^ cells, CD8^+^ cells and natural killer (NK) cells in intestinal mucosa of mice has proved the role of orally administered bLf in enhancing the host immune system. Orally administered bLf has also shown increased number of cells in lymph nodes and spleen, and enhanced production of Th1 type cytokines in systemic immune system [59]. In earlier studies, 100% iron saturated bovine Lf supplemented into the diet of mice which were challenged subcutaneously with tumor cells and treated with chemotherapy showed a decrease in the large lymphomas [13]. Along with this, 100% iron saturated bovine Lf also showed a decrease in the angiogenesis and increase in the production of Th1 and Th2 cytokines increasing apoptosis [13]. The anti-proliferative effects of bLf in cancer cells have been associated with the induction of cell cycle arrest. It has been revealed that exposure to bLf increased the cells phospho-AMPKα levels and decreased both phospho threonine mammalian target of rapamycin (mTOR) and total mTOR levels, indicating a novel mechanism of action through its ability to induce nutrient/energy-related stress in breast cancer cells [60].

Studies conducted by Tsuda group in 1997 on a colon carcinogenesis model in rats have shown a preventive effect of orally administered bLf which was further studied and confirmed on various cancer models [61,62]. A recent study has shown the inhibition of progression of colorectal polyps by administrating bLf at 3 g/day for one year. This study has shown the successful suppression of colorectal adenomas of less than 5 mm in diameter [37]. Studies involving transgenic and knockout mice have shown an increase in antimicrobial activity when Lf was administered orally [63]. Immunomodulation effect in humans was also reported with oral administration of Lf [64]. Administration of bLf results in the production of pro-inflammatory cytokine, interleukin-18 (IL-18), from the epithelial cells of the small intestine, enhancing Th1 type T and NK cell responses generating CD8+ T cells [65,66]. Inhibition of lung metastasis by bLf in B16 mice bearing the melanoma and colon cancer cells was reported by Yoo *et al*. [67] and Iigo *et al*. [37].

The effect of orally administered bLf in a randomized clinical trial [68] had shown a significant effect in retardation of polyp growth in patients with 63 years or younger age group. Increased level of human lactoferrin (hLf) in the serum of same age group was also noticed. Another randomized controlled study showed the efficacy and safety of oral bLf in combination with recombinant human erythropoietin β (rHuEPO-β) for the treatment of anemia in advanced cancer patients undergoing chemotherapy. Comparison study between erythropoiesis stimulating agents (ESAs) plus intra venous (i.v.) iron and rHuEPO-β plus lactoferrin confirmed the good efficacy of rHuEPO-β plus lactoferrin. Some of the interesting findings in this study include increased haemoglobin (Hb) level, highly significant level of iron in serum, decreased level of ferritin in rHuEPO-β plus lactoferrin and rHuEPO-β plus lactoferrin was shown very safe and without any side effects. The oral bLf administration in the hepatitis mouse model up regulated the activity of anti-inflammatory factors including IL-11 and bone morphogenetic protein 2 (BMP2). Direct production of IL-11 by bLf was also shown in the myofibroblasts on the basal side in trans well co-cultures [69]. It has also been proven from another study in 295 pregnant women suffering with iron deficiency (ID) and ID anemia (IDA) that bLf is safer and more effective in curing ID/IDA when compared to the standard ferrous sulphate therapy [70].

Oral administration of bLf is known to activate the immune system and hence is considered as a new approach for the treatment of refractory diseases including virus infection, tumor metastasis, and inflammatory bowel diseases. High amount of frequent doses of bLf is given as enzymatic hydrolysis degrades the bLf rapidly. To prevent the bLf from degradation, an appropriate delivery system is required which helps in improving the bioavailability of bLf and increases its efficiency [71].

## 3. Antimicrobial Activity of Lactoferrin

Resistance to antibiotics has led to the search of alternative therapies for sensitizing microbes. Lf has been proved to be an effective antimicrobial compound [72]. Lf has specific mechanisms by which it acts on the microorganisms. The anti-bacterial activity of Lf is attributed to its iron sequestering properties of. Lf can deplete the iron present in the system that is essential for bacterial growth and division [73]. The action of Lf on the bacterial cells is by direct contact between Lf and the bacteria. Lf is capable of directly binding to the cell wall of Gram negative bacteria, then internalizing into the cells causing iron deprivation, followed by its bacteriostatic property [74]. Lf is capable of acting on both Gram positive and Gram negative bacteria when it undergoes cleavage due to gastric enzyme pepsin. Both Lf and Lf-peptides were shown to possess antimicrobial activity. *Staphylococcus epidermidis* is one of the most predominant infectious agents in individual implemented with intraocular lenses leading to a characteristic biofilm formation on the soft contact lenses. *Staphylococcus epidermidis* can also cause severe loss of vision and cryptic infections despite the use of antibiotics like vancomycin. A study proved that, Lf was able to increase the sensitivity of this bacterium by binding to the anionic cell wall preferentially to vancomycin thereby allowing its entry into the bacteria [75]. Lf also binds to teichoic acid and increases the penetration of lysozyme by charge compensation in eye infections [76]. Another well-known example of vancomycin resistant enterococcus is *Enterococcus faecalis* Efs1. Lf has been shown to decrease d-lactate levels by increasing alanine content such that antibiotic can easily bind to the terminal alanine residue thereby hindering the formation of peptidoglycan [77]. Thus, Lf causes depolarization of the bacterial membrane leading to membrane penetration and eventually metabolic injury. Lf is also used to treat periodontal diseases by acting against plaque forming oral microorganisms like *Streptococcus mitis*, *Streptococcus gordoni*, *Streptococcus salivarius* and *Streptococcus mutans*.

Surface attachment of these microbes was compromised by Lf leading to reduced bacterial growth and biofilm formation [78]. Both hLf and bLf and their N terminal peptides were found to be more effective against Giardial infections. It has been shown that Lf was able to kill the organisms at log phase than in stationary phase in *Giardia lamblia* [79], which is better since it indicates that Lf has the ability to reduce the bacterial proliferation and growth. *Giardia lamblia* is one of the most common protozoal infections of human intestine and is also known to cause severe diarrhea. Lf produced by epithelial cells in the small intestine provides mucosal protection against this organism. A recent analysis in a cohort of children with Giardia infection fed with Lf showed a better prognosis than the unfed children. Lf supplementation decreased the degree of colonization of the species by acting on Giardia trophozoites plasmalemma, endomembrane and cytoskeleton [80]. In another study, bLf was hydrolysed using rennet and pepsin and Lactoferrin hydrolysates (LFH) were assessed for their antibacterial activities against Escherichia coli and Bacillus subtilis. The study revealed that Lf-cin B was the most potent antibacterial peptide and was isolated from both rennet and pepsin LFH [81]. The pepsin hydrolysate derivative of bLf was used to generate a bifidogenic peptide and it was found that this peptide demonstrated a stronger bifidogenic activity than natural bLf against *Bifidobacterium breve* and *Bifidobacterium longum* species [82]. Several modifications have been attempted in bLf in order to use it as a food preservative. Glycosylated lactoferrin (gLf) has been prepared using Maillard reaction and emulsifying properties, antimicrobial activities, and preservative effects on the cottage cheese in gLf was investigated. It was revealed that gLf showed substantial Fe-binding capacity and excellent emulsifying properties. It was also found that gLf completely inhibited the growth of *E. coli* at 50 °C [83]. Hence, these findings offer new possibilities for Lf as a food preservative. Nanoformulated iron saturated bLf has also been tested for its anti-microbial efficacy. Alginate gel-encapsulated ceramic nanocarriers loaded with iron-saturated bLf (AEC-CP-Fe-bLf NCs) were fed orally to BALB/c mice that were challenged with *Salmonella typhimurium* (200 μL of 10^8^ CFU/mL suspension). It was observed that nanoformulated Fe-bLf was more effective in the treatment of *Salmonella*-infected mice than the standard therapy using ciprofloxacin [18].

Anti-fungal properties of Lf are related to the capability of Lf to cause damage to the cell membrane of fungi, leading to alteration of cell membrane permeability and also its iron chelating properties [84]. Lf also exhibits antiprotozoal activity and the mechanism by which this is done varies from its antibacterial and antifungal aspects. Studies proved that although Lf had no role in inhibiting the entry of these parasites into the system but did not allow the growth of these protozoans in the host [85]. Contradictory to these effects, in some protozoans like *Trichomonas*, Lf helps in effective binding, and successful internalization in these parasites [86]. One of the most recent properties of Lf was its use as an antiviral compound. Although the research is recent, there are very few cases in which Lf failed to benefit as an antiviral activity. Lf exhibited antiviral activity against a number of viruses including herpes simplex virus, cytomegalovirus, hepatitis B and C virus (HBV and HCV) and human immunodeficiency virus (HIV) [16,87,88,89]. A new perspective in the studies of antimicrobial activity of Lf is due to its potent prophylactic and therapeutic ability in a broad spectrum. 

## 4. Lactoferrin and Immunity

**Figure 2:**
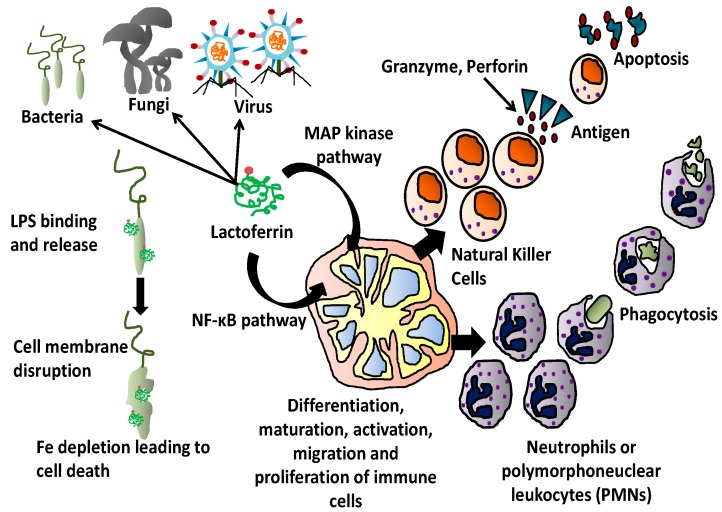
Role of lactoferrin in the activation of immune cells. Lactoferrin modulates the differentiation, maturation, activation, migration, proliferation and function of immune cells. It also promotes the cell-cell interaction and activation of PMNs and NK cells, thus boosting the immune response [96,97,98].

Apart from all the body fluids, iron free form of Lf is stored in the cytoplasmic secondary granules of neutrophils. During inflammation, Lf is released and the concentration of Lf at the site of inflammation is increased from 0.4–2.0 µg/mL to 200 µg/mL playing a major role in the feedback mechanism of inflammatory response [90]. Lf is also synthesized in the kidney supporting the immune defense system by reduction of free iron from urine and hence making it available for metabolic functions [91]. Lf is known to modulate its effect by interacting with specific cell receptors of epithelial and immune cells and as a lipopolysaccharide to pro-inflammatory bacterial elements [92,93,94]. Using two known signaling pathways, nuclear factor-kappa B (NF-κB) and MAP kinase, Lf at cellular level modulates the differentiation, maturation, activation, migration, proliferation and functions of immune cells [95]. In an *in vitro* scenario of Lf supporting the activation of immune response, at the site of injury, Lf accumulates the neutrophils which promote cell-cell interaction and activation of phagocytosis by polymorphoneuclear leukocytes (PMNs) and macrophages. As a result, the pro-inflammatory cytokines decreases in number and the activity of natural killer (NK) cells increases hence supporting the activation of lymphocytes, as shown in Figure 2 [96,97].

Lf from bovine milk showed proteinase inhibitory activity against *Porphyromonas gingivalis*, a bacterial pathogen, by inhibiting Arg- and Lys-specific proteolytic activities [99]. A clinical study investigating 472 neonates for invasive fungal infections (IFI) with very low birth weight (VLBW) showed a reduced IFI incidents in preterm VLBW neonates after the oral administration of bovine Lf [100]. The bovine Lf ingestion in ovariectomized mice showed an improved bone status via modulation in immune function. T lymphocyte activation in bone marrow showed increased number of macrophages, dendritic cells, B cells, T cells and decreased expression of TNF-α. The outcome of the study suggests the prevention of lymphocyte activation and cytokine release in the bone-marrow micro environment which is mediated via bovine Lf [101]. Lf at molecular level helps in reducing the stress and hence controls the excess inflammatory response [102]. Studies have reported that absence of Lf increases the susceptibility of inflammation. It was shown that Lf knockout mice demonstrated a great susceptibility to inflammation-induced colorectal dysplasia, mainly due to NF-κB and AKT/mTOR signaling, regulation of cell apoptosis and proliferation. These results suggest that the anti-inflammatory function of lactoferrin may contribute to its anti-tumor activity [103].

Iron plays a vital role in modulating the production of reactive oxygen species (ROS) by Haber-Weiss reaction. The production and neutralization of ROS depends on the efficiency of vital enzymes such as catalase, glutathione peroxidise and superoxide dismutase and their inefficiency can lead to over expression of hydroxyl radicals and increase in lipid peroxidation using iron-dependent Haber-Weiss reaction [104]. Endogenous Lf is hypothesized to prevent the lipid peroxidation by iron sequestration. Pre-treatment of human monocytic cells (U937) with Lf (125 and 250 µg/mL) showed a decrease in caspase 3 activity and reduction of glucose oxidase induced apoptosis. The oral intake of Lf daily was shown to support immune response via antioxidant mechanism [64]. Lf is known to play a vital role as a mediator of systemic inflammatory response syndrome by allowing the controlled regulation of inflammation without any pathological damage [95,105]. A study investigating the ability of Lf to clear the bacteria from serum and tissues of CFW mice demonstrated more than a 100- fold decrease of bacteria in circulation and in various tissues. More than 1000-fold reduction was observed in lung tissue suggesting its effective role against systemic inflammatory responses and sepsis [106,107]. Lf was also shown to increase the survival of the mice that were challenged with methicillin-resistant *Staphylococcus aureus* (MRSA) infection [108,109]. Along with the increase in survival rate, decreased IL-6 and no change in TNF-α was also demonstrated. The similar trend in both Gram-positive and Gram-negative organisms indicates the potential of Lf as a therapeutic agent during bacterial infections [110].

**Figure 3:**
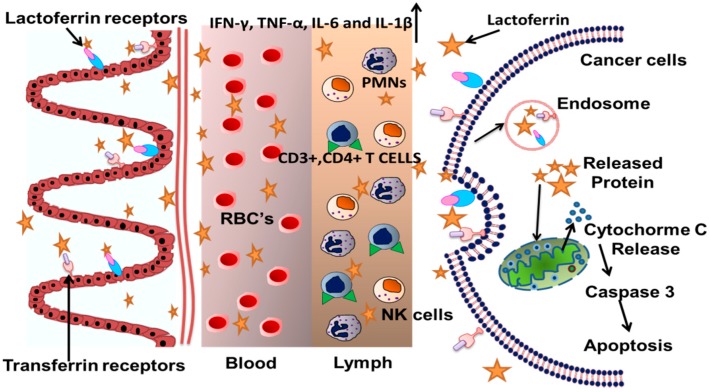
Role of lactoferrin in the activation of immune cells. Lactoferrin enters in the intestinal microvilli through the help of lactoferrin receptors and transferrin receptors present on the mucosal surface of the intestinal cells. The lactoferrin molecule further boosts up the immune response due to IFN-γ, TNF-α, IL-6 and by activating NK cells, PMNs and CD3^+^ and CD4^+^ T cells. Finally the lactoferrin enters the cells by receptor mediated endocytosis where it is released within the cells once the receptors are digested by endosomes. The lactoferrin induces release of cytochrome C from mitochondria which further activates caspase 3 to cause apoptosis in tumour cells [41,117,118,119,120,121,127].

Innate and adaptive immune response of Lf makes it an important component in first line host defense mechanism against pathogens [111,112]. High affinity towards iron makes Lf as an important component involved in nonspecific host defense system against pathogens [113]. Studies have shown the ability of Lf to induce changes in leukocytes, involved in innate immune system, by increased activity of NK cells [97,114] and increased phagocytic activity which promotes the action of neutrophils [115]. Activation of macrophages by increased production of cytokines and nitric oxide (NO) reduces the proliferation of intracellular pathogens [116,98]. The inflammatory signals degranulate the neutrophils which releases Lf into the environment comprising macrophages, dendritic cells, NK cells, T-cells and B-cells [117,118]. Increased and decreased production of pro inflammatory cytokines, TNF-α, IL-6 and IL-1β by Lf according to the requirement has been studied and examined (Figure 3).

Lf receptors present on various immune cells and the capability to bind Lf shows the specificity and potential ability of Lf to modulate the response of innate and adaptive system [119,120,121]. Activity of T cell associated with specific antigen recognition is affected by the Lf modulated antigen presenting cells (APCs). Macrophages, dendritic cells and B cells are the professional APCs, which presents the antigen to CD4^+^ T cells via major histocompatibility complex II (MHC II) [122]. Studies have shown the presence of human and bovine Lf receptors on macrophages [19]. It has been reported that, *Bacille Calmette-Guerin* strain of *Mycobacterium bovis* (BCG) led to induction of TNF-α, IL-6 and IL-1β in an infected bone marrow macrophages. These cytokines were found to be reduced by Lf. Phagocytic activity was also increased by Lf from unstimulated, LPS stimulated and infected macrophages [20,123]. Migration of Langerhan cells (LC) into the lymph node in mice was also inhibited by the recombinant Lf given 2 h prior to treatment [124]. Production of *de novo* TNF-α was also suppressed by the Lf administration resulting in the production of cytokines [125]. LPS stimulated murine bone marrow derived dendritic cells also showed decreased production of IL-12 and IL-1β when cultured in the presence of Lf [126].

All T cell subsets including δγ T cells have been reported to express Lf receptors [128]. Human Jurkat cells (T cells) have shown effective binding of both human and bovine Lf to the receptors [129,130]. Lf affects the T-cells depending upon the state of activation, differentiation and maturation [131]. hLf is known to increase the expression of human T cell ζ–chain, T cell receptor complex involved in receptor signaling [132]. Up regulation of leukocyte function associated antigen (LFA-1), an adhesion molecule present on CD4^+^ and CD8^+^ T cells, was reported in human peripheral blood mononuclear cells when cultured in presence of human Lf [133]. Decreased production of IFN-γ and IL-2 was also shown when concanavalin A (ConA) activated murine splenocytes were cultured in the presence of bovine or human Lf [134]. Oral delivery of Lf to the mice bearing tumor cells showed an increase in lymphoid and intestinal CD4^+^ and CD8^+^ T cells [135]. Increased population of circulating leukocytes CD3^+^CD4^+^, CD3^+^ TCRγδ^+^, and granulocyte were seen in mice with orally administered Lf [24]. Apart from being an immunomodulatory protein presence of lactoferrin in feces has recently been introduced as a useful tool for the diagnosis and monitoring of inflammatory bowel disease (IBD). It was revealed that fecal lactoferrin could be used to investigate or quantify the effect of granulocyte and monocyte adsorptive apheresis (GMA) in ulcerative colitis (UC) [136]. Hence, lactoferrin has multiple benefits as an immunotherapeutic and can also play a role in immunodiagnostics.

## 5. Nanoparticle-Based Cancer Therapy

The various physiological barriers in the body such as gastrointestinal environment (acidic pH in stomach) and alkaline pH in intestine are the major problems for oral supplementation of any protein [137]. A study was conducted in order to understand and determine the stability and gastric survival of Lf in the gastrointestinal tract. bLf (4.5 g, 20% Fe saturated (holoLf) and Fe unsaturated (Apo-bLf)) in the presence of a gastric pH buffer was supplemented in 12 healthy volunteers using nasogastric intubation. Using gel permeation chromatography it was revealed that 30% of Apo-bLf and 21% of holoLf were degraded by the gastric pH [138]. Hence, in order to achieve the complete payload delivery of Lf, it is necessary to protect it from the physiological barriers of the body. In the past two decades, the use of various types of nanoparticles (NPs) has shown improved delivery of anti-cancer molecules to the tumor site. Nanomedicine is the application of nanotechnology for the treatment of disease, diagnosis, monitoring, and control of biological systems [139]. The most important benefit provided by the use of NPs is protection. Many antigens/proteins/drugs/oligonucleotides are quite susceptible to degradation by various body systems such as lytic enzymes and extreme pH. NPs can shelter these antigens/proteins/drugs/oligonucleotides from this degradation. NPs also allow targeting of particular cells or body systems, contributing to efficient antigen delivery and lower dosage. It is estimated that the use of NPs could act as both carriers and adjuvants, removing the need for conventional adjuvants all together [140]. Different types of NPs (depending on their composition, shape and size) which can be taken orally for the application in cancer therapy by triggering and manipulating different signaling mechanisms, can be developed using different strategies like NP and antigen conjugation, encapsulation of antigens or peptides or DNA within NP and fluorescent labelled NPs which are identified by the receptor. The selected NPs should be drug-compatible, biocompatible, biodegradable, easy to process, have good loading efficiency and controlled drug release efficiency [141].

### 5.1. Types of Nanoparticles Used

Nanoparticles (NPs) with different compositions and distinctive physical and biological properties that might be used to overcome the limitations of molecular imaging and gene/drug delivery have been studied in recent years [142,143]. Some of the most investigated nanosystems for imaging and drug/gene delivery applications include polymeric nanoparticles, silica nanoparticles, dendrimers, quantum dots, micelles, liposomes and magnetic NPs [144,145]. Since the delivery of the native anticancer therapeutic agents to solid tumours is troublesome, drug carriers in the form of nanoparticles are an attractive alternative to target tumours and more importantly to offer limited toxicity to the normal tissues [146].

More recently, anti-angiogenic and anti-cancer nanoparticles were developed by incorporating specific antibodies and anti-cancer proteins against cancer [147]. In order to achieve specific targeting towards HER2 in breast cancer, cetuximab monoclonal antibody was either covalently [148] or non-covalently [149] coupled to liposomes, by utilizing the affinity between the binding protein coupled to cetuximab and liposomes. In both cases, specific and efficient targeting to EGFR was observed. In addition to this, modern developments in polymeric nano formulations for the treatment of cancer include a number of homo and co-polymer combinations, for sustained and controlled delivery of anti-cancer agents [150,151,152,153,154,155,156,157,158]. Polyethylene glycol-poly lactic acid polymers have been developed and successfully tested for the oral formulation of anti-angiogenic TNP470 *in vivo* [151]. Poly(lactide)–vitamin E derivative/montmorillonite nanocarrier based formulations for oral delivery of docetaxel [159] have been investigated. Various particles have been formulated for the delivery of Lf. One such example is formation of biopolymers using electrostatic complexation to form particles comprising of heat-denatured Lf particles with anionic polysaccharides (alginate, carrageenan, or pectin) [160,161,162]. Lf based particles have also been synthesized using thermal treatment where heat treatment was given to protein solution by placing it in a temperature-controlled water bath at different temperatures (70–90 °C) for different holding times (1–60 min) to create protein-based particulate-suspensions [163]. Advanced emulsions using Lf nano-particles and alginate or i-carrageenan have also been synthesized that have shown enhanced stability under *in vitro* digestive conditions [164].

### 5.2. Nanoparticle Based Therapy Using Lactoferrin 

Lf from bovine milk is one of the widely researched naturally available anti-cancer biomolecule. Previous studies have shown the anti-cancer properties of a naturally available biomolecule, Lf in its various forms (Table 2).

Decreased viability and proliferation by nearly 50% with increased apoptosis up to 2-fold by bLf has been reported in HS578T and T47D, human breast cancer cells [165]. To improve the therapeutic effects of these biomolecules, novel nanoformulations with a capability of delivering drugs with a controlled release to the target site have been evaluated [166]. The uptake of various forms of nanoparticles (NPs) has shown in different cancer models including breast cancer. Maximum cellular uptake of 15% PEGylated polylactide-co-glycolide (PLGA) NPs was recently reported in 4T1 murine breast cancer cell line [167]. Silica nanoparticles loaded with anti-HER-2 scFv800E6 antibody showed a 4-fold increase in scFv binding efficacy in HER-2 antigen-positive MCF-7 breast cancer cell line, revealing the efficiency of the nanocarrier [168].

The intraperitoneal administration of human Lf had shown the inhibition in the growth of solid tumors in mice [32]. The oral administration of bLf has also shown anti-cancer effect in the colon and other organs of the test animals [14,169]. Along with colon cancer, bLf is also known to inhibit cancer growth in oral, bladder, lung, breast, esophagus, tongue, neck and brain carcinomas [170,171]. Poly (ethyleneglycol)-poly (lactide) nanoparticles (PEG/PLA NPs) loaded with Lf showed a 3-fold increase in the amount of internalization in b.END3 brain cells compared to void NPs. With the less toxicity seen in *in vitro* and *in vivo* studies, biocompatible PEG/PLA NPs showed a promising drug delivery system [172]. Lf has also been investigated as a targeting ligand for brain specific delivery of polyamidoamine (PAMAM)-based non-viral gene vector to the brain. The transfection efficiency of PAMAM-PEG-Lf complex was 2.2-fold higher when compared to PAMAM-PEG-transferrin (Tf) complex [172,173].

**Table 2:** Various types of nanoparticles used for lactoferrin delivery.

Type of Nanoparticle	Type of Cancer	Reference
Alginate enclosed chitosan conjugated, calcium phosphate-iron-saturated bovine lactoferrin nanocarriers	Colon cancer and colon cancer stem like cells	[14]
PEG-PLGA nanoparticles	4T1 murine breast cancer	[166]
Silica nanoparticles targeted with anti-HER-2 Ab	MCF-7 breast cancer cells	[168]
PEG-PLA nanoparticles	Brain delivery	[172,173]
Super paramagnetic Iron oxide Nanoparticles	As a specific MRI contrast agent for detection of brain glioma	[174]
Biodegradable Polymersome	Chemotherapy of Glioma Rats	[175]
Procationic liposomes	Glioma	[176]
PEI/pDNA nanoparticles	Airway epithelial cells	[177]
Polyamidoamine (PAMAM) conjugated with lactoferrin	Rotenone-induced chronic Parkinson model	[178]
Chitosan/alginate/calcium complex micro particles loaded with lactoferrin	Carrageenan-induced edema in rats	[179]
Lf modified DNA loaded nanoparticles	Brain capillary endothelial cells	[180]
Alginate enclosed chitosan coated calcium phosphate nanoparticles	Colon cancer	[181]
Paclitaxel loaded Lf coupled solid lipid nanoparticles (SLPs)	Human bronchial epithelial cells (BEAS-2B)	[182]
Folic acid and Lf functionalized PLGA nanoparticles loaded with etoposide	Glioblastoma	[183]

In addition to the targeted delivery, research is also focusing on imaging using imaging contrast agents as NPs. Supermagnetic iron oxide nanoparticles conjugated with Lf (Lf-SPIONs) were studied for specific delivery and imaging in a rat model of C6 glioma. High expression of Lf receptors in brain tumor tissue compared to normal brain tissue showed the sensitivity of Lf-SPIONs towards glioma. Along with more accumulation of Lf-SPIONs in brain, the better picture quality on magnetic resonance (MRI) images suggested Lf-SPIONs as a potential and sensitive MRI contrast agent for the diagnosis of brain glioma [174]. Biodegradable polymersome encapsulated with doxorubicin and tetrandrine and conjugated with Lf (Lf-PO-Dox/Tet) showed a strong cytotoxic effect against C6 glioma cells. The maximum accumulation was observed at the tumor site in brain, the treatment reduced tumor size and increased survival rate in Lf-PO-Dox/Tet treatment group [175]. Lf modified doxorubicin loaded procationic liposomes were also studied as a brain targeted chemotherapeutic delivery system. Improved uptake efficiency of the NPs in primary brain capillary endothelial cells and C6 glioma cells was reported. More efficient inhibition in the growth of C6 glioma *in vitro*, suggested Lf modified doxorubicin loaded procationic liposomes as an effective therapeutic formulation to treat glioma compared to other doxorubicin formulations [176]. A study investigating Lf as a targeting ligand for receptor mediated gene delivery to human bronchial epithelial cells, suggested Lf as a potent targeting ligand and a promising candidate for *in vivo* lung gene transfer [177]. The intravenous administration of Lf modified polyamidoamine (PAMAM) NPs loaded with human glial cell line-derived neurotrophic factor gene (hGDNF), showed improved locomotor activity, reduced dopaminergic neuronal loss and increased monoamine neurotransmitter levels in rats with rotenone induced Parkinson’s disease [178]. Chitosan/alginate/calcium microparticles loaded with 20%–30% (w/w) Lf showed a suppressive effect against carrageenan-induced edema in rats [179]. A study investigating Lf modified DNA loaded NPs showed the internalization by brain capillary endothelial cells (BCECs) via clathrin-dependent endocytosis, caveolae-mediated endocytosis, and macropinocytosis [180]. Recently alginate enclosed chitosan coated calcium phosphate NPs loaded with iron saturated bLf (Fe-bLf) with spherical morphology showed an increased and efficient anti-cancer efficiency *in vitro* without affecting mucosal integrity during transcytosis. The oral administration of these NPs given one week prior to injecting colon cancer in mice showed high efficiency without any toxicity. Orally given Fe-bLf in nanoformulation after the development of tumor in mice showed significant reduction in tumor size and complete rejection within 35 days compared to void NPs [181]. In another study, alginate-enclosed, chitosan-conjugated, calcium phosphate-iron-saturated bovine lactoferrin (Fe-bLf) nanocarriers/nanocapsules (NCs) showed effective internalization and reduction of cancer stem cell markers in triple-positive CD133, survivin and CD44 cancer stem-like cells both *in vitro* and *in vivo*. It was found that the serum iron, zinc and calcium absorption were increased. DMT1, LRP, transferrin and lactoferrin receptors were responsible for internalization of the NCs. The study also revealed that The NCs activated both extrinsic, as well as intrinsic apoptotic pathways to induce apoptosis by targeting survivin in cancer cells and cancer stem cells, without inducing any nonspecific nanotoxicity [14]. Recently, it has been shown that LF receptors can be utilized for the transportation of Lf-conjugated drug or nanocarrier devices. Paclitaxel (PTX)-loaded Lf-coupled solid lipid nanoparticles (SLNs) were evaluated for their anti-cancer potential by *in vitro* (human bronchial epithelial BEAS-2B cells), *ex vivo* and *in vivo* evaluations. In vivo biodistribution studies showed higher concentrations of PTX accumulated in lungs via Lf-coupled SLNs than plain SLNs and free PTX [182]. Another recent study used Lf and folic acid (FA) on poly(lactide-co-glycolide) (PLGA) nanoparticles (NPs) for transporting etoposide across the blood-brain barrier (BBB) and treating human brain malignant glioblastoma [183].

Apart from cancer, Lf nanoparticles have been used for arthritis therapy as well. An injectable hydrogel was formed using bLf, cross linking agent tyramine, horse radish peroxidase and hydrogen peroxide (H_2_O_2_). The hydrogel showed promising ability to decrease osteoclastogenesis and increase osteoblast proliferation in an *in vitro* model [184]. In another study, the therapeutic potentials of 100% iron saturated-bovine lactoferrin encapsulated in alginate-chitosan polymeric nanocarriers (AEC-CP-Fe-bLf-NCs) were examined in *in vitro* inflammatory osteoarthritis (OA) model and in collagen-induced arthritis (CIA) mice. It was revealed that these NCs showed highly promising anti-inflammatory properties and induced reversal of OA by dissoluting the calcium pyrophosphate crystals found in mice joints [38].

## 6. Lf in Clinical Trials

Over 1300 peer-reviewed medical and scientific journals have put forth the inherent ability of Lf as anti-cancer, anti-viral, anti-bacterial and as anti-fungal agent. The multifunctional role of bLf has been established for over twenty-five years now [185]. This astounding research captivated many pharmaceuticals to hover around this protein. Fonterra, a leading multinational diary company and the world’s largest exporter of dairy products has produced “Recharge” ice-cream made of Lf. Phase II clinical trial of this innovative ice-cream is expected to enhance the immune system in patients undergoing chemotherapy. Iron saturated Lf from Fonterra, also enhanced the production of Th1, Th2, IFN-γ and TNF thereby sensitizing the tumors to chemotherapy *in vitro* [13]. A very recent study on Lf led to the discovery of curing iron deficiency in complicated pregnancies with improved Hb levels. bLf restores the physiological transport of iron from tissues to circulation thus enhancing iron homeostasis. Pregnant women are at high risk of iron deficiency since the developing fetus requires an enormous content of iron for its development. Hepicidin, a master regulator of iron homeostasis helps to increase iron storage in cells. Lf administration decreased IL-6 concentration and increased pro-hepcidin in pregnant women while in non-pregnant women; Lf did not change IL-6 levels while it increased pro-hepcidin [186]. A novel beverage that contains iron saturated Lf to fortify iron has been shown to improve hematocrit (Ht) levels, RBC number and Hb levels [187]. Lf was also effective in modulating blood glucose levels by increasing insulin content in diabetes mellitus patients [21]. Thus a vast clinical need for an effective treatment can be envisaged by the use of this multifunctional protein.

## 7. Conclusions

The versatility of Lf has been the focus of this review. The advantages of this natural molecule prove its potential as a natural therapeutic agent that can be used in various fields of research including cancer. The role of Lf as anti-bacterial and anti-fungal agent had been beneficial in its use as a bactericidal and fungicidal agent in lotions and creams. Its use can be extended to topical applications as well. An interesting aspect of using Lf as an anti-cancer agent by delivering it to the body in the form of ice-creams, tablets and oral supplements in the form of NPs have been researched upon. With its role in being able to combat deadly viruses like HCV and HBV also poses a need for its use as an anti-viral agent for human immunodeficiency virus (HIV) and other potent viruses that cause health risks. The role of this natural molecule as anti-inflammatory agent needs further research. It stands as a biomarker for inflammatory conditions and its potential role as a therapeutic molecule needs to be taken forward.

## References

[1] Nagpal R., Behare P., Rana R., Kumar A., Kumar M., Arora S., Morotta F., Jain S., Yadav H. (2011). Bioactive peptides derived from milk proteins and their health beneficial potentials: An update. Food Funct..

[2] Baker E., Baker H. (2005). Lactoferrin. Cell. Mol. Life Sci..

[3] Iigo M., Alexander D.B., Long N., Xu J., Fukamachi K., Futakuchi M., Takase M., Tsuda H. (2009). Anticarcinogenesis pathways activated by bovine lactoferrin in the murine small intestine. Biochimie.

[4] Birgens H.S. (1985). Lactoferrin in plasma measured by an elisa technique: Evidence that plasma lactoferrin is an indicator of neutrophil turnover and bone marrow activity in acute leukaemia. Scand. J. Haematol..

[5] Mastromarino P., Capobianco D., Campagna G., Laforgia N., Drimaco P., Dileone A., Baldassarre M.E. (2014). Correlation between lactoferrin and beneficial microbiota in breast milk and infant’s feces. Biometals.

[6] Parhi P., Mohanty C., Sahoo S.K. (2012). Nanotechnology-based combinational drug delivery: An emerging approach for cancer therapy. Drug Discov. Today.

[7] Sanchez L., Calvo M., Brock J.H. (1992). Biological role of lactoferrin. Arch. Dis. Child..

[8] González-Chávez S.A., Arévalo-Gallegos S., Rascón-Cruz Q. (2009). Lactoferrin: Structure, function and applications. Int. J. Antimicrob. Agents.

[9] Iafisco M., Foggia M.D., Bonora S., Prat M., Roveri N. (2011). Adsorption and spectroscopic characterization of lactoferrin on hydroxyapatite nanocrystals. Dalton Trans..

[10] Aisen P., Leibman A. (1972). Lactoferrin and transferrin: A comparative study. BBA-Protein Struct..

[11] Wakabayashi H., Yamauchi K., Takase M. (2006). Lactoferrin research, technology and applications. Int. Dairy J..

[12] Burrow H., K Kanwar R., R Kanwar J. (2011). Antioxidant enzyme activities of iron-saturated bovine lactoferrin (Fe-blf) in human gut epithelial cells under oxidative stress. Med. Chem..

[13] Kanwar J.R., Palmano K.P., Sun X., Kanwar R.K., Gupta R., Haggarty N., Rowan A., Ram S., Krissansen G.W. (2008). Iron-saturated lactoferrin is a potent natural adjuvant for augmenting cancer chemotherapy. Immunol. Cell Biol..

[14] Kanwar J.R., Mahidhara G., Roy K., Sasidharan S., Krishnakumar S., Prasad N., Sehgal R., Kanwar R.K. (2014). Fe-blf nanoformulation targets survivin to kill colon cancer stem cells and maintains absorption of iron, calcium and zinc. Nanomedicine.

[15] Hiss S., Meyer T., Sauerwein H. (2008). Lactoferrin concentrations in goat milk throughout lactation. Small Rumin. Res..

[16] Hara K., Ikeda M., Saito S., Matsumoto S., Numata K., Kato N., Tanaka K., Sekihara H. (2002). Lactoferrin inhibits hepatitis b virus infection in cultured human hepatocytes. Hepatol. Res..

[17] Agrawal R.P., Dogra R., Mohta N., Tiwari R., Singhal S., Sultania S. (2009). Beneficial effect of camel milk in diabetic nephropathy. Acta Biomed..

[18] Gupta I., Sehgal R., Kanwar R.K., Punj V., Kanwar J.R. (2014). Nanocapsules loaded with iron-saturated bovine lactoferrin have antimicrobial therapeutic potential and maintain calcium, zinc and iron metabolism. Nanomedicine.

[19] Roseanu A., Chelu F., Trif M., Motas C., Brock J.H. (2000). Inhibition of binding of lactoferrin to the human promonocyte cell line thp-1 by heparin: The role of cell surface sulphated molecules. Biochim. Biophys. Acta.

[20] Wilk K.M., Hwang S.A., Actor J.K. (2007). Lactoferrin modulation of antigen-presenting-cell response to bcg infection. Postepy Hig. Med. Dosw..

[21] Mohamad R.H., Zekry Z.K., Al-Mehdar H.A., Salama O., El-Shaieb S.E., El-Basmy A.A., Al-said M.G., Sharawy S.M. (2009). Camel milk as an adjuvant therapy for the treatment of type 1 diabetes: Verification of a traditional ethnomedical practice. J. Med. Food.

[22] Tsuda H., Ohshima Y., Nomoto H., Fujita K.I., Matsuda E., Iigo M., Takasuka N., Moore M.A. (2004). Cancer prevention by natural compounds. Drug Metabol. Pharmacokinet..

[23] Zhang Y., Lima C.F., Rodrigues L.R. (2014). Anticancer effects of lactoferrin: Underlying mechanisms and future trends in cancer therapy. Nutr. Rev..

[24] Wakabayashi H., Takakura N., Yamauchi K., Tamura Y. (2006). Modulation of immunity-related gene expression in small intestines of mice by oral administration of lactoferrin. Clin. Vaccine Immunol..

[25] Reitamo S., Konttinen Y., Segerberg-Konttinen M. (1980). Distribution of lactoferrin in human salivary glands. Histochemistry.

[26] McClellan K. (1997). Mucosal defense of the outer eye. Surv. Ophthalmol..

[27] Valore E.V., Park C.H., Igreti S.L., Ganz T. (2002). Antimicrobial components of vaginal fluid. Am. J. Obstet. Gynecol..

[28] Breton-Gorius J., Mason D., Buriot D., Vilde J., Griscelli C. (1980). Lactoferrin deficiency as a consequence of a lack of specific granules in neutrophils from a patient with recurrent infections. Detection by immunoperoxidase staining for lactoferrin and cytochemical electron microscopy. Am. J. Pathol..

[29] Masson P.L., Heremans J.F., Schonne E. (1969). Lactoferrin, an iron-binding protein in neutrophilic leukocytes. J. Exp. Med..

[30] Caccavo D., Garzia P., Sebastiani G.D., Ferri G.M., Galluzzo S., Vadacca M., Rigon A., Afeltra A., Amoroso A. (2003). Expression of lactoferrin on neutrophil granulocytes from synovial fluid and peripheral blood of patients with rheumatoid arthritis. J. Rheumatol..

[31] Arnold R.R., Brewer M., Gauthier M.M. (2001). Bactericidal activity of human lactoferrin: Sensitivity of a variety of microorganisms. Infect. Immun..

[32] Bezault J., Bhimani R., Wiprovnick J., Firmanski P. (1994). Human lactoferrin inhibits growth of solid tumors and development of metastasis in mice. Cancer Res..

[33] Nichols B.L., Mckee K.S., Henry J.F., Putman M. (1987). Human lactoferrin stimulates thymidine incorporation into DNA of rat crypt cells. Pediat. Res..

[34] Uchida K., Matsuse R., Tomiota S., Sugi K., Saitoh O., Ohshiba S. (1994). Immunochemical detection of human lactoferrin in feces as a new marker for inflammatory gastrointestinal disorders and colon cancer. Clin. Biochem..

[35] Redwan E.R.M., Tabll A. (2007). Camel lactoferrin markedly inhibits hepatitis c virus genotype 4 infection of human peripheral blood leukocytes. J. Immun. Immunochem..

[36] Nozaki A., Tanaka K., Naganuma A., Kato N. (2002). Recent advances of basic research and clinical application of lactoferrin as an antiviral reagent against chronic hepatitis c. Nippon Rinsho.

[37] Iigo M., Alexander D.B., Xu J., Futakuchi M., Suzui M., Kozu T., Akasu T., Saito D., Kakizoe T., Yamauchi K. (2014). Inhibition of intestinal polyp growth by oral ingestion of bovine lactoferrin and immune cells in the large intestine. Biometals.

[38] Samarasinghe R.M., Kanwar R.K., Kanwar J.R. (2014). The effect of oral administration of iron saturated-bovine lactoferrin encapsulated chitosan-nanocarriers on osteoarthritis. Biomaterials.

[39] Crouch S.P., Slater K.J., Fletcher J. (1992). Regulation of cytokine release from mononuclear cells by the iron-binding protein lactoferrin. Blood.

[40] Yamaguchi M., Matsuura M., Kobayashi K., Sasaki H., Yajima T., Kuwata T. (2001). Lactoferrin protects against development of hepatitis caused by sensitization of kupffer cells by lipopolysaccharide. Clin. Diagn. Lab. Immunol..

[41] Haversen L., Ohlsson B.G., Hahn-Zoric M., Hanson L.A., Mattsby-Baltzer I. (2002). Lactoferrin down-regulates the LPS-induced cytokine production in monocytic cells via NF-kappa b. Cell. Immunol..

[42] Sugiyama A., Sato A., Shimizu H., Ando K., Takeuchi T. (2010). Pegylated lactoferrin enhances its hepatoprotective effects on acute liver injury induced by d-galactosamine and lipopolysaccharide in rats. J. Vet. Med. Sci..

[43] Cole M.F., Arnold R.R., Mestecky J., Prince S., Kulhavy R., McGhee J.R., Stiles M., Loesche W.J., O’Brien T.C. (1977). Studies with human lactoferrin and s. Mutans. Microbial Aspects of Dental Caries.

[44] Le Parc A., Dallas D.C., Duaut S., Leonil J., Martin P., Barile D. (2014). Characterization of goat milk lactoferrin n-glycans and comparison with the N-glycomes of human and bovine milk. Electrophoresis.

[45] El-Fakharany E.M., Tabil A., El-Wahab A.A., Haroun B.M., Redwan E. (2008). Potential activity of camel milk-amylase and lactoferrin against hepatitis c virus infectivity in hepg2 and lymphocytes. Hepat. Mon..

[46] Bonkovsky H.L., Banner B.F., Rothman A.L. (1997). Iron and chronic viral hepatitis. Hepatology.

[47] Farinati F., Cardin R., Maria N.D., Libera G.D., Marafin C., Lecis E. (1995). Iron storage, lipid peroxidation and glutathione turnover in chronic anti-HCV positive hepatitis. J. Hepatol..

[48] Khan J.A., Kumar P., Paramasivam M., Yadav R.S., Sahani M.S., Sharma S., Srinivasan A., Singh T.P. (2001). Camel lactoferrin, a transferrin-cum-lactoferrin crystal structures of camel apolactoferrin at a 2.6 Å resolution and structural basis of its dual role. J. Mol. Biol..

[49] Konishi M.I., Yamauchi K., Sugimoto R., Fujita N., Kobayashi Y., Watanabe S. (2006). Lactoferrin inhibits lipid peroxidation in patients with chronic hepatitis c. Hepatol. Res..

[50] El-Agamy E.I., Park Y.W. (2009). Bioactive components in camel milk. Bioactive Components in Milk and Dairy Products.

[51] Saltanat H., Li H., Xu Y., Wang J., Liu F., Geng H.H. (2009). The influences of camel milk on the immune response of chronic hepatitis b patients. Milk Res..

[52] Al-Hashem F., Dallak M., Bashir N., Abbas M. (2009). Camel’s milk protects against cadmium chloride induced toxicity in white albino rats. Am. J. Pharmacol. Toxicol..

[53] Bellamy W., Takase M., Yamauchi K., Wakabayashi H., Kawase K., Tomita M. (1992). Identification of the bactericidal domain of lactoferrin. BBA-Protein Struct. Mol. Enzymol..

[54] Chierici R., Sawatzki G., Tamisari L., Volpato S., Vigi V. (1992). Supplementation of an adapted formula with bovine lactoferrin. 2. Effects on serum iron, ferritin and zinc levels. Acta Paediatr..

[55] King J.C., Cummings G.E., Guo N., Trivedi L., Readmond B.X., Keane V., Feigelman S., de Waard R. (2007). A double-blind, placebo-controlled, pilot study of bovine lactoferrin supplementation in bottle-fed infants. J. Pediat. Gastroenterol. Nutr..

[56] Takakura N., Wakabayashi H., Ishibashi H., Yamauchi K., Teraguchi S., Tamura Y., Yamaguchi H., Abe S. (2004). Effect of orally administered bovine lactoferrin on the immune response in the oral candidiasis murine model. J. Med. Microbiol..

[57] Shin K., Wakabayashi H., Yamauchi K., Teraguchi S., Tamura Y., Kurokawa M., Shiraki K. (2005). Effects of orally administered bovine lactoferrin and lactoperoxidase on influenza virus infection in mice. J. Med. Microbiol..

[58] Marchetti M., Pisani S., Antonini G., Valenti P., Seganti L., Orsi N. (1998). Metal complexes of bovine lactoferrin inhibit *in vitro* replication of herpes simplex virus type 1 and 2. Biometals.

[59] Fischer R., Debbabi H., Dubarry M., Boyaka P., Tome D. (2006). Regulation of physiological and pathological th1 and th2 responses by lactoferrin this paper is one of a selection of papers published in this special issue, entitled 7th international conference on lactoferrin: Structure, function, and applications, and has undergone the journal's usual peer review process. Biochem. Cell Biol..

[60] Zhang Y., Nicolau A., Lima C.F., Rodrigues L.R. (2014). Bovine lactoferrin induces cell cycle arrest and inhibits mtor signaling in breast cancer cells. Nutr. Cancer.

[61] Sekine K., Watanabe E., Nakamura J., Takasuka N., Kim D.J., Asamoto M., Krutovskikh V., Baba-Toriyama H., Ota T., Moore M.A. (1997). Inhibition of azoxymethane-initiated colon tumor by bovine lactoferrin administration in f344 rats. Cancer Sci..

[62] Ushida Y., Sekine K., Kuhara T., Takasuka N., Iigo M., Maeda M., Tsuda H. (1999). Possible chemopreventive effects of bovine lactoferrin on esophagus and lung carcinogenesis in the rat. Cancer Sci..

[63] Takakura N., Wakabayashi H., Ishibashi H., Teraguchi S., Tamura Y., Yamaguchi H., Abe S. (2003). Oral lactoferrin treatment of experimental oral candidiasis in mice. Antimicrob. Agents Chemother..

[64] Mulder A.M., Connellan P.A., Oliver C.J., Morris C.A., Stevenson L.M. (2008). Bovine lactoferrin supplementation supports immune and antioxidant status in healthy human males. Nutr. Res..

[65] Okamoto I., Kohno K., Tanimoto T., Ikegami H., Kurimoto M. (1999). Development of CD8+ effector T cells is differentially regulated by IL-18 and IL-12. J. Immunol..

[66] Iigo M.M., Kuhara T.T., Ushida Y.Y., Sekine K.K., Moore M.A.M., Tsuda H.H. (1999). Inhibitory effects of bovine lactoferrin on colon carcinoma 26 lung metastasis in mice. Clin. Exp. Metastas..

[67] Yoo Y.C., Watanabe S., Watanabe R., Hata K., Shimazaki K.I., Azuma I. (1997). Bovine lactoferrin and lactoferricin, a peptide derived from bovine lactoferrin, inhibit tumor metastasis in mice. Cancer Sci..

[68] Macciò A. (2010). Efficacy and safety of oral lactoferrin supplementation in combination with rhuepo-β for the treatment of anemia in advanced cancer patients undergoing chemotherapy: Open-label, randomized controlled study. Oncologist.

[69] Kuhara T., Yamauchi K., Iwatsuki K. (2012). Bovine lactoferrin induces interleukin-11 production in a hepatitis mouse model and human intestinal myofibroblasts. Eur. J. Nutr..

[70] Paesano R., Pacifici E., Benedetti S., Berlutti F., Frioni A., Polimeni A., Valenti P. (2014). Safety and efficacy of lactoferrin *versus* ferrous sulphate in curing iron deficiency and iron deficiency anaemia in hereditary thrombophilia pregnant women: An interventional study. Biometals.

[71] Onishi H. (2011). Lactoferrin delivery systems: Approaches for its more effective use. Expert Opin. Drug Deliv..

[72] Li Y.M., Tan A.X., Vlassara H. (1995). Antibacterial activity of lysozyme and lactoferrin is inhibited by binding of advanced glycation-modified proteins to a conserved motif. Nature Med..

[73] Bullen J.J., Rogers H.J., Leigh L. (1972). Iron binding proteins in milk and resistance to escherichia coli infection in infants. Br. Med. J..

[74] Ellison R.T., Giehl T.J., la Force F.M. (1988). Damage of the outer membrane of enteric gram-negative bacteria by lactoferrin and transferrin. Infect. Immun..

[75] Leitch E.C., Willcox M.D. (1999). Lactoferrin increases the susceptibility of s. Epidermidis biofilms to lysozyme and vancomycin. Curr. Eye Res..

[76] Leitch E.C., Willcox M.D. (1999). Elucidation of the antistaphylococcal mechanism of lactoferrin and lysozyme. J. Med. Microbiol..

[77] Leitch E.C., Willcox M.D. (2000). Lactoferrin induced reduction of vanb vancomycin resistance in enterococci. Int. J. Antimicrob. Agents.

[78] Roseanu A., Florian P., Condei M., Cristea D., Damian M. (2010). Antibacterial activity of lactoferrin and lactoferricin against oral streptococci. Rom. Biotechnol. Lett..

[79] Turchany J.M., Aley S.B., Gillin F.D. (1995). Giardicidal activity of lactoferrin and N-terminal peptides. Infect. Immun..

[80] Ochoa T.J., Woo E.W., Campos M., Pecho M., Prada A., McMahon R.J., Cleary T.G. (2008). Impact of lactoferrin supplementation on growth and prevalence of giardia colonization in children. Clin. Infect. Dis..

[81] Elbarbary H.A., Abdou A.M., Park E.Y., Nakamura Y., Mohamed H.A., Sato K. (2010). Novel antibacterial lactoferrin peptides generated by rennet digestion and autofocusing technique. Int. Dairy J..

[82] Oda H., Wakabayashi H., Yamauchi K., Sato T., Xiao J.Z., Abe F., Iwatsuki K. (2013). Isolation of a bifidogenic peptide from the pepsin hydrolysate of bovine lactoferrin. Appl. Environ. Microbiol..

[83] Nakamura K. (2002). Potent antimicrobial effects of the glycosylated lactoferrin. Food Preserv. Sci..

[84] Wakabayashi H., Uchida K., Yamauchi K., Teraguchi S., Hayasawa H., Yamaguchi H. (2000). Lactoferrin given in food facilitates dermatophytosis cure in guinea pig models. J. Antimicrob. Chemother..

[85] Cintra W.M., Silva-Filho F.C., de Souza W. (1986). The surface charge of toxoplasma gondii: A cytochemical and electrophoretic study. J. Submicrosc. Cytol..

[86] Tachezy J., Suchan P., Schrevel J., Kulda J. (1998). The host-protein independent iron uptake by tritrichomonas foetus. Exp. Parasitol..

[87] Ikeda M., Sugiyama K., Tanaka T., Tanaka K., Sekihara H., Shimotohno K., Kato N. (1998). Lactoferrin markedly inhibits hepatitis c virus infection in cultured human hepatocytes. Biochem. Biophys. Res. Commun..

[88] Harmsen M.C., Swart P.J., de Bethune M.P., Pauwels R., de Clercq E., The T.H., Meijer D.K. (1995). Antiviral effects of plasma and milk proteins: Lactoferrin shows potent activity against both human immunodeficiency virus and human cytomegalovirus replication *in vitro*. J. Infect. Dis..

[89] Roy K., Kanwar R.K., Kanwar J.R. (2012). Targeting viral hepatitis using natural milk protein and traditional medicinal herbs. J. Clin. Cell. Immunol..

[90] Farnaud S., Evans R.W. (2003). Lactoferrin—A multifunctional protein with antimicrobial properties. Mol. Immunol..

[91] Abrink M., Larsson E., Gobl A., Hellman L. (2000). Expression of lactoferrin in the kidney: Implications for innate immunity and iron metabolism. Kidney Int..

[92] Na Y.J., Han S.B., Kang J.S., Yoon Y.D., Park S.K., Kim H.M., Yang K.H., Joe C.O. (2004). Lactoferrin works as a new LPS-binding protein in inflammatory activation of macrophages. Int. Immunopharmacol..

[93] Elass-Rochard E., Roseanu A., Legrand D., Trif M., Salmon V., Motas C., Montreuil J., Spik G. (1995). Lactoferrin-lipopolysaccharide interaction: Involvement of the 28–34 loop region of human lactoferrin in the high-affinity binding to *Escherichia coli* 055b5 lipopolysaccharide. Biochem. J..

[94] Legrand D., Pierce A., Elass E., Carpentier M., Mariller C., Mazurier J., Bösze Z. (2008). Lactoferrin Structure and Functions Bioactive Components of Milk.

[95] Gahr M., Speer C., Damerau B., Sawatzki G. (1991). Influence of lactoferrin on the function of human polymorphonuclear leukocytes and monocytes. J. Leukoc. Biol..

[96] Damiens E., Mazurier J., El Yazidi I., Masson M., Duthille I., Spik G., Boilly-Marer Y. (1998). Effects of human lactoferrin on nk cell cytotoxicity against haematopoietic and epithelial tumour cells. BBA-Mol. Cell Res..

[97] Dashper S.G., Pan Y., Veith P.D., Chen Y.Y., Toh E.C., Liu S.W., Cross K.J., Reynolds E.C. (2012). Lactoferrin inhibits porphyromonas gingivalis proteinases and has sustained biofilm inhibitory activity. Antimicrob. Agents Chemother..

[98] Kawai K., Shimazaki K., Higuchi H., Nagahata H. (2007). Antibacterial activity of bovine lactoferrin hydrolysate against mastitis pathogens and its effect on superoxide production of bovine neutrophils. Zoonoses Public Health.

[99] Manzoni P., Stolfi I., Messner H., Cattani S., Laforgia N., Romeo M.G., Bollani L., Rinaldi M., Gallo E., Quercia M. (2012). Bovine lactoferrin prevents invasive fungal infections in very low birth weight infants: A randomized controlled trial. Pediatrics.

[100] Malet A., Bournaud E., Lan A., Mikogami T., Tome D., Blais A. (2011). Bovine lactoferrin improves bone status of ovariectomized mice via immune function modulation. Bone.

[101] Touyz R.M. (2000). Oxidative stress and vascular damage in hypertension. Curr. Hypertens. Rep..

[102] Ye Q., Zheng Y., Fan S., Qin Z., Li N., Tang A., Ai F., Zhang X., Bian Y., Dang W. (2014). Lactoferrin deficiency promotes colitis-associated colorectal dysplasia in mice. PLoS ONE.

[103] Gutteridge J., Richmond R., Halliwell B. (1979). Inhibition of the iron-catalysed formation of hydroxyl radicals from superoxide and of lipid peroxidation by desferrioxamine. Biochem. J..

[104] Raetz C.R., Ulevitch R.J., Wright S.D., Sibley C.H., Ding A., Nathan C.F. (1991). Gram-negative endotoxin: An extraordinary lipid with profound effects on eukaryotic signal transduction. FASEB J..

[105] Pajkrt D., Doran J.E., Koster F., Lerch P.G., Arnet B., van der Poll T., ten Cate J.W., van Deventer S.J. (1996). Antiinflammatory effects of reconstituted high-density lipoprotein during human endotoxemia. J. Exp. Med..

[106] Zagulski T., Lipinski P., Zagulska A., Broniek S., Jarzabek Z. (1989). Lactoferrin can protect mice against a lethal dose of *Escherichia coli* in experimental infection *in vivo*. Br. J. Exp. Pathol..

[107] Zagulski T., Lipinski P., Zagulska A., Jarzabek Z. (1998). Antibacterial system generated by lactoferrin in mice *in vivo* is primarily a killing system. Int. J. Exp. Pathol..

[108] Foster T.J. (2005). Immune evasion by staphylococci. Nat. Rev. Microbiol..

[109] Holtfreter S., Broker B.M. (2005). Staphylococcal superantigens: Do they play a role in sepsis?. Arch. Immunol. Ther. Exp..

[110] Ellison R. (1991). Killing of gram-negative bacteria by lactoferrin and lysozyme. J. Clin. Investig..

[111] Legrand D., Elass E., Carpentier M., Mazurier J. (2005). Lactoferrin: A modulator of immune and inflammatory responses. Cell. Mol. Life Sci..

[112] Kruzel M.L., Harari Y., Mailman D., Actor J.K., Zimecki M. (2002). Differential effects of prophylactic, concurrent and therapeutic lactoferrin treatment on LPS-induced inflammatory responses in mice. Clin. Exp. Immunol..

[113] Baynes R.D., Bezwoda W.R. (1994). Lactoferrin and the inflammatory response. Adv. Exp. Med. Biol..

[114] Shau H., Kim A., Golub S.H. (1992). Modulation of natural killer and lymphokine-activated killer cell cytotoxicity by lactoferrin. J. Leukoc. Biol..

[115] Miyauchi H., Hashimoto S., Nakajima M., Shinoda I., Fukuwatari Y., Hayasawa H. (1998). Bovine lactoferrin stimulates the phagocytic activity of human neutrophils: Identification of its active domain. Cell. Immunol..

[116] Wakabayashi H., Takakura N., Teraguchi S., Tamura Y. (2003). Lactoferrin feeding augments peritoneal macrophage activities in mice intraperitoneally injected with inactivated candida albicans. Microbiol. Immunol..

[117] Suzuki Y.A., Lopez V., Lonnerdal B. (2005). Mammalian lactoferrin receptors: Structure and function. Cell. Mol. Life Sci..

[118] Hammarstrom M.L., Mincheva-Nilsson L., Hammarstrom S. (1995). Functional lactoferrin receptors on activated human lymphocytes. Adv. Exp. Med. Biol..

[119] Sorimachi K., Akimoto K., Hattori Y., Ieiri T., Niwa A. (1997). Activation of macrophages by lactoferrin: Secretion of tnf-alpha, il-8 and no. Biochem. Mol. Biol. Int..

[120] Zimecki M., Wlaszczyk A., Wojciechowski R., Dawiskiba J., Kruzel M. (2001). Lactoferrin regulates the immune responses in post-surgical patients. Arch. Immunol. Ther. Exp..

[121] Machnicki M., Zimecki M., Zagulski T. (1993). Lactoferrin regulates the release of tumour necrosis factor alpha and interleukin 6 *in vivo*. Int. J. Exp. Pathol..

[122] Puddu P., Valenti P., Gessani S. (2009). Immunomodulatory effects of lactoferrin on antigen presenting cells. Biochimie.

[123] Tanida T., Rao F., Hamada T., Ueta E., Osaki T. (2001). Lactoferrin peptide increases the survival of candida albicans-inoculated mice by upregulating neutrophil and macrophage functions, especially in combination with amphotericin b and granulocyte-macrophage colony-stimulating factor. Infect. Immun..

[124] Cumberbatch M., Dearman R.J., Uribe-Luna S., Headon D.R., Ward P.P., Conneely O.M., Kimber I. (2000). Regulation of epidermal langerhans cell migration by lactoferrin. Immunology.

[125] Cumberbatch M., Bhushan M., Dearman R.J., Kimber I., Griffiths C.E. (2003). IL-1beta-induced langerhans’ cell migration and tnf-alpha production in human skin: Regulation by lactoferrin. Clin. Exp. Immunol..

[126] Mikkelsen T.L., Bakman S., Sorensen E.S., Barkholt V., Frokiaer H. (2005). Sialic acid-containing milk proteins show differential immunomodulatory activities independent of sialic acid. J. Agric. Food Chem..

[127] Zimecki M., Dawiskiba J., Zawirska B., Krawczyk Z., Kruzel M. (2003). Bovine lactoferrin decreases histopathological changes in the liver and regulates cytokine production by splenocytes of obstructive jaundiced rats. Inflamm. Res..

[128] Mincheva-Nilsson L., Hammarstrom S., Hammarstrom M.L. (1997). Activated human gamma delta t lymphocytes express functional lactoferrin receptors. Scand. J. Immunol..

[129] Tanaka T., Morita H., Yoo Y.C., Kim W.S., Kumura H., Shimazaki K. (2004). Detection of bovine lactoferrin binding protein on jurkat human lymphoblastic T cell line. J. Vet. Med. Sci..

[130] Legrand D., van Berkel P.H., Salmon V., van Veen H.A., Slomianny M.C., Nuijens J.H., Spik G. (1997). The *N*-terminal arg2, arg3 and arg4 of human lactoferrin interact with sulphated molecules but not with the receptor present on jurkat human lymphoblastic T-cells. Biochem. J..

[131] Zimecki M., Mazurier J., Machnicki M., Wieczorek Z., Montreuil J., Spik G. (1991). Immunostimulatory activity of lactotransferrin and maturation of CD4- CD8- murine thymocytes. Immunol. Lett..

[132] Frydecka I., Zimecki M., Bocko D., Kosmaczewska A., Teodorowska R., Ciszak L., Kruzel M., Wlodarska-Polinsk J., Kuliczkowski K., Kornafel J. (2002). Lactoferrin-induced up-regulation of zeta (zeta) chain expression in peripheral blood T lymphocytes from cervical cancer patients. Anticancer Res..

[133] Zimecki M., Miedzybrodzki R., Mazurier J., Spik G. (1999). Regulatory effects of lactoferrin and lipopolysaccharide on lfa-1 expression on human peripheral blood mononuclear cells. Arch. Immunol. Ther. Exp..

[134] Sfeir R.M., Dubarry M., Boyaka P.N., Rautureau M., Tomé D. (2004). The mode of oral bovine lactoferrin administration influences mucosal and systemic immune responses in mice. J. Nutr..

[135] Wang W.-P., Iigo M., Sato J., Sekine K., Adachi I., Tsuda H. (2000). Activation of intestinal mucosal immunity in tumor-bearing mice by lactoferrin. Cancer Sci..

[136] Hashiguchi K., Takeshima F., Akazawa Y., Matsushima K., Minami H., Machida H., Yamaguchi N., Shiozawa K., Ohba K., Ohnita K. (2015). Advantages of fecal lactoferrin measurement during granulocyte and monocyte adsorptive apheresis therapy in ulcerative colitis. Digestion.

[137] Furlund C., Ulleberg E., Devold T., Flengsrud R., Jacobsen M., Sekse C., Holm H., Vegarud G. (2013). Identification of lactoferrin peptides generated by digestion with human gastrointestinal enzymes. J. Dairy Sci..

[138] Troost F.J., Steijns J., Saris W.H., Brummer R.J.M. (2001). Gastric digestion of bovine lactoferrin *in vivo* in adults. J. Nutr..

[139] Liu Y., Miyoshi H., Nakamura M. (2007). Nanomedicine for drug delivery and imaging: A promising avenue for cancer therapy and diagnosis using targeted functional nanoparticles. Int. J. Cancer.

[140] Kawasaki E.S., Player A. (2005). Nanotechnology, nanomedicine, and the development of new, effective therapies for cancer. Nanomedicine.

[141] Lü J.M., Wang X., Marin-Muller C., Wang H., Lin P.H., Yao Q., Chen C. (2009). Current advances in research and clinical applications of plga-based nanotechnology. Expert Rev. Mol. Diagn..

[142] Koo O.M., Rubinstein I., Onyuksel H. (2005). Role of nanotechnology in targeted drug delivery and imaging: A concise review. Nanomedicine.

[143] Shapira A., Livney Y.D., Broxterman H.J., Assaraf Y.G. (2011). Nanomedicine for targeted cancer therapy: Towards the overcoming of drug resistance. Drug Resist. Updates.

[144] Mitra A., Nan A., Line B.R., Ghandehari H. (2006). Nanocarriers for nuclear imaging and radiotherapy of cancer. Curr. Pharm. Des..

[145] Zrazhevskiy P., Sena M., Gao X. (2010). Designing multifunctional quantum dots for bioimaging, detection, and drug delivery. Chem. Soc. Rev..

[146] Xie J., Lee S., Chen X. (2010). Nanoparticle-based theranostic agents. Adv. Drug Deliv. Rev..

[147] Yoncheva K., Momekov G. (2011). Antiangiogenic anticancer strategy based on nanoparticulate systems. Expert Opin. Drug Deliv..

[148] Mamot C., Drummond D.C., Greiser U., Hong K., Kirpotin D.B., Marks J.D., Park J.W. (2003). Epidermal growth factor receptor (egfr)-targeted immunoliposomes mediate specific and efficient drug delivery to egfr- and egfrviii-overexpressing tumor cells. Cancer Res..

[149] Pan X., Lee R.J. (2007). Construction of anti-egfr immunoliposomes via folate-folate binding protein affinity. Int. J. Pharm..

[150] Hwang H.Y., Kim I.S., Kwon I.C., Kim Y.H. (2008). Tumor targetability and antitumor effect of docetaxel-loaded hydrophobically modified glycol chitosan nanoparticles. J. Control. Release.

[151] Benny O., Fainaru O., Adini A., Cassiola F., Bazinet L., Adini I., Pravda E., Nahmias Y., Koirala S., Corfas G. (2008). An orally delivered small-molecule formulation with antiangiogenic and anticancer activity. Nat. Biotechnol..

[152] Kim J.H., Kim Y.S., Park K., Kang E., Lee S., Nam H.Y., Kim K., Park J.H., Chi D.Y., Park R.W. (2008). Self-assembled glycol chitosan nanoparticles for the sustained and prolonged delivery of antiangiogenic small peptide drugs in cancer therapy. Biomaterials.

[153] Zhang L., Sun M., Guo R., Jiang Z., Liu Y., Jiang X., Yang C. (2006). Chitosan surface-modified hydroxycamptothecin loaded nanoparticles with enhanced transport across caco-2 cell monolayer. J. Nanosci. Nanotechnol..

[154] Balakrishnan P., Lee B.J., Oh D.H., Kim J.O., Lee Y.I., Kim D.D., Jee J.P., Lee Y.B., Woo J.S., Yong C.S. (2009). Enhanced oral bioavailability of coenzyme Q10 by self-emulsifying drug delivery systems. Int. J. Pharm..

[155] Wang A., Li S. (2008). Hydroxycamptothecin-loaded nanoparticles enhance target drug delivery and anticancer effect. BMC Biotechnol..

[156] Howard K.A., Paludan S.R., Behlke M.A., Besenbacher F., Deleuran B., Kjems J. (2008). Chitosan/sirna nanoparticle-mediated tnf-[alpha] knockdown in peritoneal macrophages for anti-inflammatory treatment in a murine arthritis model. Mol. Ther..

[157] Yadav S., van Vlerken L., Little S., Amiji M. (2009). Evaluations of combination mdr-1 gene silencing and paclitaxel administration in biodegradable polymeric nanoparticle formulations to overcome multidrug resistance in cancer cells. Cancer Chemother. Pharmacol..

[158] Zhang Z., Lee S., Gan C., Feng S.S. (2008). *In vitro* and *in vivo* investigation on pla–tpgs nanoparticles for controlled and sustained small molecule chemotherapy. Pharm. Res..

[159] Feng S.S., Mei L., Anitha P., Gan C.W., Zhou W. (2009). Poly(lactide)-vitamin E derivative/montmorillonite nanoparticle formulations for the oral delivery of docetaxel. Biomaterials.

[160] Peinado I., Lesmes U., Andrés A., McClements J.D. (2010). Fabrication and morphological characterization of biopolymer particles formed by electrostatic complexation of heat treated lactoferrin and anionic polysaccharides. Langmuir.

[161] Bengoechea C., Jones O.G., Guerrero A., McClements D.J. (2011). Formation and characterization of lactoferrin/pectin electrostatic complexes: Impact of composition, pH and thermal treatment. Food Hydrocolloids.

[162] David-Birman T., Mackie A., Lesmes U. (2013). Impact of dietary fibers on the properties and proteolytic digestibility of lactoferrin nano-particles. Food Hydrocoll..

[163] Bengoechea C., Peinado I., McClements D.J. (2011). Formation of protein nanoparticles by controlled heat treatment of lactoferrin: Factors affecting particle characteristics. Food Hydrocoll..

[164] Shimoni G., Levi C.S., Tal S.L., Lesmes U. (2013). Emulsions stabilization by lactoferrin nano-particles under *in vitro* digestion conditions. Food Hydrocoll..

[165] Duarte D.C., Nicolau A., Teixeira J.A., Rodrigues L.R. (2011). The effect of bovine milk lactoferrin on human breast cancer cell lines. J. Dairy Sci..

[166] Paliwal S.R., Paliwal R., Agrawal G.P., Vyas S.P. (2011). Liposomal nanomedicine for breast cancer therapy. Nanomedicine.

[167] Pamujula S., Hazari S., Bolden G., Graves R.A., Chinta D.D., Dash S., Kishore V., Mandal T.K. (2012). Cellular delivery of pegylated plga nanoparticles. J Pharm. Pharmacol..

[168] Mazzucchelli S., Verderio P., Sommaruga S., Colombo M., Salvadè A., Corsi F., Galeffi P., Tortora P., Prosperi D. (2011). Multiple presentation of scfv800e6 on silica nanospheres enhances targeting efficiency toward her-2 receptor in breast cancer cells. Bioconj. Chem..

[169] Tsuda H., Kozu T., Iinuma G., Ohashi Y., Saito Y., Saito D., Akasu T., Alexander D., Futakuchi M., Fukamachi K. (2010). Cancer prevention by bovine lactoferrin: From animal studies to human trial. Biometals.

[170] Sakai T., Banno Y., Kato Y., Nozawa Y., Kawaguchi M. (2005). Pepsin-digested bovine lactoferrin induces apoptotic cell death with jnk/sapk activation in oral cancer cells. J. Pharmacol. Sci..

[171] Tsuda H., Sekine K., Fujita K.I., Iigo M. (2002). Cancer prevention by bovine lactoferrin and underlying mechanisms—A review of experimental and clinical studies. Biochem. Cell Biol..

[172] Hu K., Li J., Shen Y., Lu W., Gao X., Zhang Q., Jiang X. (2009). Lactoferrin-conjugated PEG–PLA nanoparticles with improved brain delivery: *In vitro* and *in vivo* evaluations. J. Control. Release.

[173] Huang R., Ke W., Liu Y., Jiang C., Pei Y. (2008). The use of lactoferrin as a ligand for targeting the polyamidoamine-based gene delivery system to the brain. Biomaterials.

[174] Xie H., Zhu Y., Jiang W., Zhou Q., Yang H., Gu N., Zhang Y., Xu H., Xu H., Yang X. (2010). Lactoferrin-conjugated superparamagnetic iron oxide nanoparticles as a specific MRI contrast agent for detection of brain glioma *in vivo*. Biomaterials.

[175] Pang Z., Feng L., Hua R., Chen J., Gao H., Pan S., Jiang X., Zhang P. (2010). Lactoferrin-conjugated biodegradable polymersome holding doxorubicin and tetrandrine for chemotherapy of glioma rats. Mol. Pharm..

[176] Chen H., Qin Y., Zhang Q., Jiang W., Tang L., Liu J., He Q. (2011). Lactoferrin modified doxorubicin-loaded procationic liposomes for the treatment of gliomas. Eur. J. Pharm. Sci..

[177] Elfinger M., Maucksch C., Rudolph C. (2007). Characterization of lactoferrin as a targeting ligand for nonviral gene delivery to airway epithelial cells. Biomaterials.

[178] Huang R., Ke W., Liu Y., Wu D., Feng L., Jiang C., Pei Y. (2010). Gene therapy using lactoferrin-modified nanoparticles in a rotenone-induced chronic parkinson model. J. Neurol. Sci..

[179] Onishi H., Koyama K., Sakata O., Machida Y. (2010). Preparation of chitosan/alginate/calcium complex microparticles loaded with lactoferrin and their efficacy on carrageenan-induced edema in rats. Drug Dev. Ind. Pharm..

[180] Huang R., Ke W., Han L., Liu Y., Shao K., Ye L., Lou J., Jiang C., Pei Y. (2009). Brain-targeting mechanisms of lactoferrin-modified DNA-loaded nanoparticles. J. Cereb. Blood Flow Metabol..

[181] Kanwar J.R., Mahidhara G., Kanwar R.K. (2012). Novel alginate-enclosed chitosan-calcium phosphate-loaded iron-saturated bovine lactoferrin nanocarriers for oral delivery in colon cancer therapy. Nanomedicine.

[182] Pandey V., Gajbhiye K.R., Soni V. (2015). Lactoferrin-appended solid lipid nanoparticles of paclitaxel for effective management of bronchogenic carcinoma. Drug Deliv..

[183] Kuo Y.C., Chen Y.C. (2015). Targeting delivery of etoposide to inhibit the growth of human glioblastoma multiforme using lactoferrin-and folic acid-grafted poly (lactide-co-glycolide) nanoparticles. Inter. J. Pharm..

[184] Amini A.A., Kan H.M., Cui Z., Maye P., Nair L.S. (2014). Enzymatically cross-linked bovine lactoferrin as injectable hydrogel for cell delivery. Tissue Eng. Part A.

[185] Tomita M., Wakabayashi H., Shin K., Yamauchi K., Yaeshima T., Iwatsuki K. (2009). Twenty-five years of research on bovine lactoferrin applications. Biochimie.

[186] Paesano R., Berlutti F., Pietropaoli M., Goolsbee W., Pacifici E., Valenti P. (2010). Lactoferrin efficacy *versus* sulphate in curing iron disorders in pregnant and non-pregnant women. Int. J. Immunopathol. Pharmacol..

[187] Tanaka M.S., Tojima T., Dousako S., Tatsumi K. (1992). Method of Manufacturing Iron-Fortified Beverage. U.S. Patent.

